# Recurrence following Resection of Intraductal Papillary Mucinous Neoplasms: A Systematic Review to Guide Surveillance

**DOI:** 10.3390/jcm13030830

**Published:** 2024-01-31

**Authors:** Aneesa Salahuddin, Varna Thayaparan, Ahmad Hamad, Willi Tarver, Jordan M. Cloyd, Alex C. Kim, Robyn Gebhard, Timothy M. Pawlik, Bradley N. Reames, Aslam Ejaz

**Affiliations:** 1 Department of Surgery, The Ohio State University, Columbus, OH 43210, USA; aneesa1011@gmail.com (A.S.); jordan.cloyd@osumc.edu (J.M.C.); tim.pawlik@osumc.edu (T.M.P.); 2 Department of Internal Medicine, The Ohio State University, Columbus, OH 43210, USA; 3 Department of Radiology, The Ohio State University, Columbus, OH 43210, USA; 4 Department of Surgery, University of Nebraska Medical Center, Omaha, NE 68198, USA; 5 Department of Surgery, University of Ilinois at Chicago, 840 S. Wood Street, Chicago, IL 60612, USA

**Keywords:** follow-up, progression, IPMN, recurrence-free survival, pancreas cyst, pancreatic cystic neoplasm, pancreatectomy

## Abstract

Patients who undergo resection for non-invasive IPMN are at risk for long-term recurrence. Further evidence is needed to identify evidence-based surveillance strategies based on the risk of recurrence. We performed a systematic review of the current literature regarding recurrence patterns following resection of non-invasive IPMN to summarize evidence-based recommendations for surveillance. Among the 61 studies reviewed, a total of 8779 patients underwent resection for non-invasive IPMN. The pooled overall median follow-up time was 49.5 months (IQR: 38.5–57.7) and ranged between 14.1 months and 114 months. The overall median recurrence rate for patients with resected non-invasive IPMN was 8.8% (IQR: 5.0, 15.6) and ranged from 0% to 27.6%. Among the 33 studies reporting the time to recurrence, the overall median time to recurrence was 24 months (IQR: 17, 46). Existing literature on recurrence rates and post-resection surveillance strategies for patients with resected non-invasive IPMN varies greatly. Patients with resected non-invasive IPMN appear to be at risk for long-term recurrence and should undergo routine surveillance.

## 1. Background

Pancreatic Intraductal Papillary Mucinous Neoplasms (IPMN) are a type of pre-malignant cystic neoplasm that develops within the pancreatic duct. The incidence of IPMN has been increasing in recent years, likely due to an increase in the use of cross-sectional imaging for other causes [1]. This has led to some authors to classify IPMNs as part of a larger group of “Pancreatic Incidentalomas” [2]. As a premalignant lesion, IPMN can be detected across a range of different stages of the neoplastic spectrum, ranging from low-grade dysplasia to invasive carcinoma.

While modern techniques to diagnose IPMN have improved in recent years, risk-stratifying the malignant potential of these lesions remains challenging. Several consensus-based guidelines exist to help guide the management of these complex cystic neoplasms [2,3]. As the overwhelming majority of IPMN have a benign course and morbidity of pancreatic resection remains high, risk-based surveillance constitutes the mainstay of management. Despite this, there remains a lack of accurate pre-operative staging for IPMNs, as nearly one-half of patients who undergo resection for IPMNs harbor only low-grade dysplasia [2,3]. For patients with IPMN that harbor “worrisome” or “high-risk stigmata” for malignancy, surgical resection remains the primary treatment.

For patients who undergo surgical resection for IPMN, the optimal frequency and type of post-resection surveillance remains unclear. Previous studies hypothesized IPMN as a “field defect”, suggesting that the entire pancreatic gland remains at risk of developing recurrent IPMN or invasive carcinoma even after complete surgical resection of the index IMPN [4]. Recurrent disease necessitating repeat surgical resection has been reported to be as high as 62% during long-term (10-year) follow-up [4]. For patients found to have invasive carcinoma after resection of IPMN, post-resection surveillance strategies are based on established rates and patterns of recurrences. Yet, there is significant heterogeneity in consensus recommendations for surveillance for patients found to have either no, low-grade, or high-grade dysplasia. For example, the American Gastroenterological Association (AGA) recommends against routine surveillance after the resection of IPMN without invasive malignancy or high-grade dysplasia, whereas European guidelines recommend that all patients with IPMN should undergo lifetime surveillance after surgery [5]. Whether the degree of dysplasia, or other clinicopathologic features, should influence these strategies remains unknown. As a result, surveillance strategies are highly variable in practice largely determined by individual practice patterns.

In the context of conflicting data and disparate consensus-based recommendations, we aimed to systematically review the current literature regarding recurrence patterns following resection of non-invasive IPMN to summarize evidence-based recommendations for surveillance.

## 2. Methods

### 2.1. Literature Search Strategy

We conducted a comprehensive search strategy in the MEDLINE database for studies published between January 2000 through January 2022. The following keywords and Medical Subject Headings were included in our search: “IPMN” or “Intraductal Papillary Mucinous Neoplasm” and “follow-up” or “surveillance” or “recurrence” or “progression”. The references of relevant articles were also reviewed to identify additional eligible publications. The methodology utilized the standards of the Preferred Reporting Items for Systematic Reviews and Meta-Analyses (PRISMA). This systematic review was not registered.

### 2.2. Design and Study Selection

Two researchers (AS, VT) independently reviewed all potentially eligible studies for inclusion in the review. The titles and abstracts of the identified studies were screened. When deemed necessary, full-texts of relevant articles were retrieved and carefully assessed against the eligibility criteria. Studies were eligible for inclusion if they reported on the recurrence or surveillance patterns among patients who underwent resection for initially non-invasive IPMN. The exclusion criteria were as follows: (1) case studies, (2) studies including patients with other cystic neoplasms that were not IPMN, (3) studies lacking data about recurrence/progression rates, (4) reports that examined patients who had unconfirmed IPMN or did not undergo surgical resection as the primary treatment, and (5) non-empirical studies such as conference abstracts that did not proceed to publication in peer-reviewed journals. Only studies available in English were considered eligible.

### 2.3. Data Extraction

Data regarding type of study, type of IPMN, type of surgical resection, follow-up time, frequency and type of surveillance, and recurrence/progression rates were collected from each paper. When multiple studies analyzed the same population (i.e., series from the same hospital), data were extracted from the larger study or the study with longer follow-up time. To identify such studies, we assessed each study’s setting (name of hospital, university affiliation, and location) and time period, as well as each study’s investigators.

### 2.4. Statistical Analysis

Summary statistics were reported as total and percentage for categorical variables and as median values and interquartile ranges unless stated otherwise for continuous variables. The results were not pooled into a meta-analysis due to the variation and substantial heterogeneity among the included studies. Two reviewers (AS, VT) compiled the data for data synthesis and analysis.

## 3. Results

### 3.1. Literature Search and Study Selection

**Figure 1:**
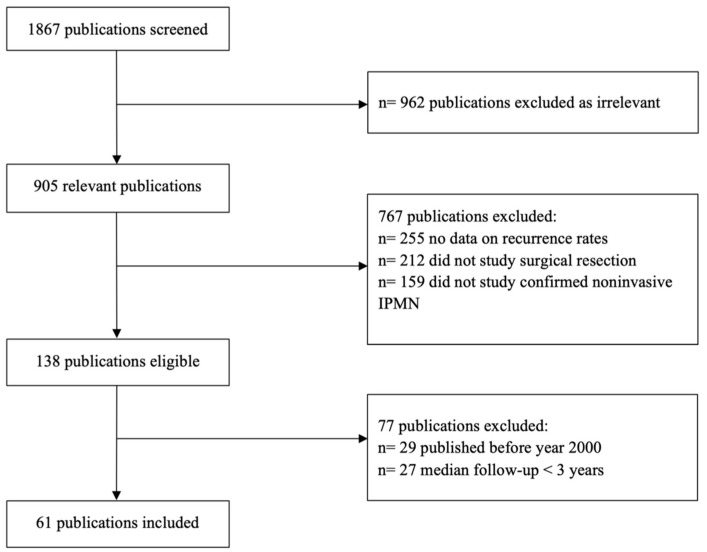
Flow chart of literature search and study selection.

A total of 1867 studies were identified using the MEDLINE database (Figure 1). After evaluation, 905 were selected for further review after excluding 962 studies that were not related to the study topic. A further 762 publications were excluded as these studies focused primarily on surveillance without data on surgical resection, were case studies, had patients with presumed or non-confirmed IPMN, or did not include data on IPMN recurrence. Moreover, 77 studies were excluded as these articles were either published before the year 2000, focused on other cystic neoplasms with less than 10 cases of IPMN, or had median follow-up time shorter than 3 months. This screening process yielded 61 studies that were included in our review for analysis (Figure 1) [4,5,6,7,8,9,10,11,12,13,14,15,16,17,18,19,20,21,22,23,24,25,26,27,28,29,30,31,32,33,34,35,36,37,38,39,40,41,42,43,44,45,46,47,48,49,50,51,52,53,54,55,56,57,58,59,60,61,62,63,64,65,66]. Among the 61 studies included, 44 were retrospective in nature (72.1%) and the remaining 17 (27.9%) were prospective.

### 3.2. Baseline Characteristics

Overall, a total of 9733 patients with clinical, radiological, or pathological diagnosis of IPMN were included; 8779 patients underwent resection for non-invasive disease (Table 1). 20 studies (32.8%) were published between 2000–2009, including patient data from 1979–2006. 41 studies (67.2%) were published in 2010 or later, including patient data from 1987–2020. Among the 61 studies included, sample sizes ranged from as few as 15 patients to the largest retrospective cohort study that included 827 patients [57]. Most studies included patients with branch duct, main duct, and mixed-type IPMN (*n* = 39), whereas 4 studies (6.6%) included patients with only main duct IPMN; 5 studies included patients with only branch duct IPMN (8.2%), and 1 study (1.6%) included patients with only mixed-type IPMN. The remaining 12 studies (19.7%) included various combinations of two IPMN types (main and branch duct or main and mixed-type).

### 3.3. Type of Resection

Overall, 21 studies reported the type and number of each operation performed. Patients included in these studies most commonly underwent a pancreaticoduodenectomy (*n* = 2385, 59.5%), followed by a distal pancreatectomy (*n* = 1119, 27.9%), total pancreatectomy (*n* = 245, 6.1%), central pancreatectomy (*n* = 180, 4.5%), and other procedures (*n* = 83, 2.0%).

### 3.4. Patterns of Post-Resection Surveillance

**Figure 2:**
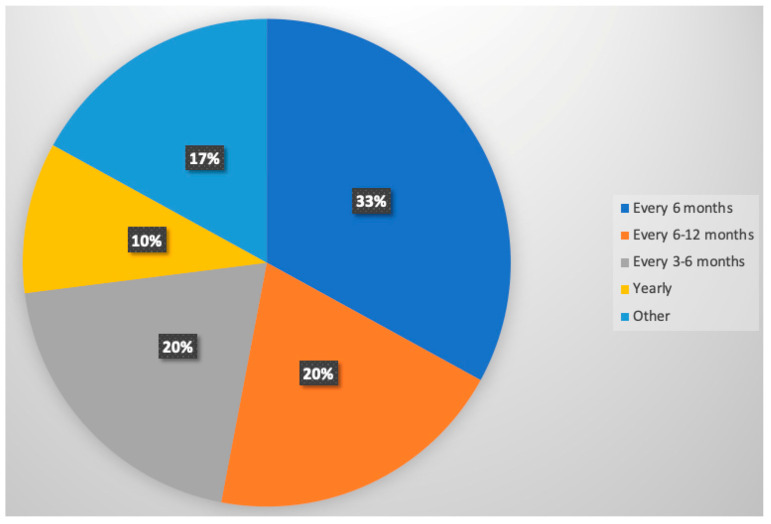
Frequencies and patterns of post-resection surveillance strategies.

The pooled overall median follow-up time for patients with resected non-invasive IPMN was 49.5 months (IQR: 38.5–57.7). Median follow-up times ranged between 14.1 months and 114 months. Of the 61 studies included in this review, 30 reported details on the intensity of post-resection surveillance for patients with non-invasive IPMN (Table 2). The most commonly reported frequency of post-resection surveillance was every 6 months (*n* = 10, 33%), followed by every 6 to 12 months (*n* = 6, 20%), every 3 to 6 months (*n* = 6, 20%), and yearly (*n* = 3, 10%) (Figure 2). Two studies had more specific post-resection surveillance schedules such as every 3 months for the first year, every 6 months for the second year, and yearly thereafter [63] or more intense surveillance after 2 years depending on tumor invasiveness [31]. One study by Yamaguchi et al. reported 3-month post-resection surveillance for the first 2 years followed by every 6 months after. Kwon et al. reported post-resection surveillance schedules of 1, 3, and 6 months for the first year, then yearly afterwards.

Forty-two studies reported the imaging modalities used for post-resection surveillance. The most common modality used was a combination of computed tomography (CT) and magnetic resonance imaging (MRI) (*n* = 15, 35.7%). This was followed by a combination of CT/MRI and ultrasound (US) imaging (either endoscopic or abdominal) (*n* = 13, 31.0%), CT imaging alone (*n* = 10, 23.8%), or a combination of CT and US (*n* = 2, 4.8%). Several studies reported alternative imaging modalities including positron emission tomography (PET) scan (*n* = 1, 2.4%), magnetic resonance cholangiopancreatography (*n* = 5, 11.9%), or endoscopic resonance cholangiopancreatography imaging (*n* = 2, 4.8%). Three studies (7.1%) reported using blood tumor markers as a means of surveillance after surgical resection.

### 3.5. Recurrence Rates and Patterns

The definition of “recurrence” was not explicitly defined in the majority of studies. However, for studies that define define recurrence, it was identified as a new IPMN or cancer in the remnant gland following resection. The overall median recurrence rate for patients with resected non-invasive IPMN was 8.8% (IQR: 5.0, 15.6). Reported recurrence rates ranged from 0% to 27.6%. Among the 33 studies reporting the time to recurrence, the overall median time to recurrence was 24 months (IQR: 17, 46) (Table 3). Among papers reporting mean or average time to recurrence, the overall mean time to recurrence was 40.8 months (SD, 29.4). Of the 61 studies included in this study, only 8 studies reported recurrence rates with location of recurrence. Among a total of 1380 patients from these 8 studies, 130 (9.4%) patients had a recurrence of a non-invasive IPMN recurrence, 63 (4.6%) patients developed PDAC, and 5 (0.4%) patients developed metastatic PDAC disease.

### 3.6. Disease-Free and Overall Survival

Six studies reported disease-free survival (DFS) at 1-year. The overall 1-year DFS was 95.1% (IQR: 90.0, 97.8). 5-year DFS was 88% (IQR: 82.5, 94) among twenty-seven studies and 10-year DFS was 78% (IQR: 72.8–86.1) among seven studies. Only 5 studies reported overall survival (OS) rates with a cumulative OS of 92% (IQR: 81, 92) at 5 years.

## 4. Discussion

Patients who undergo resection for non-invasive IPMN remain at risk for developing recurrent IPMN and/or PDAC [4]. To our knowledge, this is the largest systematic review of recurrence rates and post-resection surveillance for patients who have undergone resection for non-invasive IPMN. With advances in imaging technology in recent years, and the increased incidence of IPMN, the current study is important to help guide long-term management for patients with non-invasive IPMN. In the current study, recurrence rates varied greatly and ranged from 0% to 27.6%. Furthermore, the reporting of recurrence rates was non-uniform across studies. These data have important implications that can help guide and standardize post-resection surveillance schedules as well as standardize the reporting of future studies related to long-term outcomes among patients with resected non-invasive IPMN.

In the current study, the pooled median recurrence rate was 8.8% (IQR, 5.0, 15.6) with a median time to recurrence of 24 months (IQR, 17–46) for all patients who underwent resection for non-invasive IPMN. These findings are in line with previously reported recurrence rates ranging between 1% and 20% [27,65,67]. The definition of recurrence may even, in fact, vary across studies, as some investigators may not consider a new IPMN as a true “recurrence”, but rather as a de facto new lesion within the remnant pancreas and introduce bias into the findings [68]. Despite this lack of a clear definition, previous studies have indicated that patients are at risk of recurrence even beyond 10 years post-resection [4]. The current median follow-up time in the current study was only 49.5 months; therefore, the incidence of recurrence may have been under-represented as recurrences likely occurred beyond this time period. There are several proposed factors that may increase the risk of post-resection recurrence and help guide post-resection surveillance patterns such as IPMN with low- or high-grade dysplasia, a margin-positive resection, certain genetic mutations (i.e., *SMAD4, TP53* etc.), and having a family history of PDAC [2]. Despite the likelihood of recurrence, it does appear that salvage treatment may be possible as long-term overall survival remains high at 92% at 5 years. Furthermore, it is important to note that recurrence rates in the included studies reported recurrences of IPMN as well as PDAC. However, the reported recurrence rates of invasive PDAC after resection of non-invasive IPMN were less than 1% among the included studies.

There are numerous consensus-based guidelines that offer recommendations for the management of post-resection surveillance for patients with non-invasive IPMN [2,3,69,70,71]. However, these guidelines are largely based off low-quality evidence and consensus statements and vary greatly in their type and frequency of surveillance schedules. For instance, guidelines from the American College of Radiology do not comment on any type of post-resection surveillance whereas the Fukuoka guidelines recommend lifetime cross-sectional imaging surveillance until patients are no longer surgical candidates [2,72]. In the current systematic review, there was significant variation in the frequency of post-resection surveillance utilized. Furthermore, the type of surveillance also varied greatly. For instance, most studies reported using CT or MRI, however several studies utilized ultrasonography and endoscopic ultrasonography for surveillance.

Based on the pooled recurrence rates and patterns found in the current study, we recommend cross-sectional imaging every 6–12 months for all patients with resected IPMN. Future research should focus on identifying biomarkers and other features that allow for tailored risk-based surveillance. Similarly, based on the likelihood of recurrence, as well as the possibility of long-term recurrence, we recommend lifelong post-resection surveillance, as long as the patient remains a surgical candidate. Novel treatment strategies for IPMN such as chemotherapeutic and thermal ablation argue further in favor of lifelong follow-up regardless of the ability to undergo pancreatic resection.

There are several limitations of this systematic review largely due to the quality of evidence and heterogeneity of the studies included. The retrospective nature of most of the studies included introduces potential selection bias. Moreover, details regarding follow-up frequencies, recurrence rates, and median or average time to recurrence were missing from numerous studies and highlights the need for more standardized reporting of IPMN outcomes. Additionally, several studies did not stratify according to non-invasive or invasive disease and thus were unable to be included in the current review. Due to these limitations, we did not pool results of the included studies statistically, preventing us from carrying out a meta-analysis. These limitations highlight the need for future prospective trials to generate sufficient high-quality evidence to guide practice for these patients.

## 5. Conclusions

In conclusion, existing literature on recurrence rates and post-resection surveillance strategies for patients with resected non-invasive IPMN varies greatly. Patients with resected non-invasive IPMN appear to be at risk for long-term recurrence and should undergo routine surveillance. Future work should focus on creating evidence-based standardized recommendations to guide patient surveillance.

## Figures and Tables

**Table 1:** Included Studies.

Author	Publication Year	Years Patient Data Collected	Non-Invasive IPMN Sample Size *n*
Marchegiani	2015	1990–2013	299
Al Efishat	2018	1989–2015	319
Marchegiani	2015	1990–2013	106
Pflüger	2020	1995–2009	124
Amini	2020	1995–2018	449
Li	2020	2013–2019	125
Kim	2020	2000–2018	431
Kwon	2019	2005–2016	253
Dhar	2018		330
Jang	2016	2004–2014	74
Yogi	2015	1988–2014	153
Xourafas	2015	2002–2012	87
Kang	2014	1995–2013	298
Frankel	2013	1990–2010	192
Winner	2013	1994–2011	183
Passot	2012	1994–2009	104
Cheon	2010	1998–2008	25
Landa	2009	1996–2006	67
Nagai	2007	1984–2006	42
Yokoyama	2007	1979–2005	100
Takahashi	2006	1992–2004	20
Chari	2002	1983–2002	60
Hirono	2020	1996–2014	827
D’Angelica	2004	1983–2000	32
Fujii	2010	1991–2009	81
Salvia	2004	1988–2002	140
Schnelldorfer	2008	1992–2005	143
White	2007	1983–2006	78
Blair	2021	2004–2016	127
Sugimachi	2021	2005–2020	25
Majumder	2019	1997–2014	138
Asano	2020	1990–2019	85
Nagai	2019	2004–2016	74
Poruk	2019	1997–2016	546
Antoñanzas	2018	1993–2016	18
Date	2018	1987–2015	135
Pea	2017	1996–2014	260
Kimura	2017	1994–2015	71
Marsoner	2016		24
Ridtitid	2016	2001–2013	117
Hirono	2016	1999–2014	172
Miyasaka	2016	1987–2012	160
Yamaguchi	2016	2004–2013	40
Kwon	2014	1995–2013	19
Sahora	2014	1993–2012	43
Sauvanet	2014	1999–2011	75
Tamura	2014	1987–2012	36
Yuan	2014	2001–2011	24
Sahora K	2013	1995–2012	203
Distler M	2013	1995–2010	33
He	2013		130
Ohtsuka	2012		136
Miller	2011		191
Fujii	2011		84
Park	2011	1995–2009	68
Nakagohri	2010	1994–2007	13
Crippa	2010	1988–2006	389
Lubezky	2010	2002–2008	39
Niedergethmann	2008	1996–2006	29
Rodriguez	2007	1990–2005	113
Cuillerier	2000	1980–1996	20

**Table 2:** Post-resection surveillance strategies.

Author	Follow-Up Frequency	Imaging at Follow-Up
Al Efishat	6–12 months	CT/MRI
Pflüger	6 months	CT, MRI, & PET/CT
Amini	6–12 months	CT, MRI, or EUS
Li	6–12 months	CT, MRI, EUS; serum tumor markers
Kim	every 3 months for 1st year, 6 months for 2nd year, then yearly	CT, MRI
Kwon	3–6 months for invasive, not given for noninvasive	CT or MRI
Jang	6–12 months	Ultrasonography or CT
Yogi	6 months	Contrast-enhanced CT
Kang	3 months (first year), 6 months (second year), subsequent depended on tumor invasiveness	CT, MRI
Winner	3–6 months	MRI, CT, or EUS
Passot	At least yearly (dependent on invasiveness)	CT, MRI
Cheon	Seen at 6 and 12 months, then yearly	CT
Yokoyama	3–6 months	US, CT, or MRI (ERCP, EUS, and IDUS used to confirm if recurrence was suspected)
Takahashi	3–6 months	Abdominal US, CT, MRI
Hirono	3–6 months	CT, MRI, EUS
Fujii	6 months	CT/MDCT or EUS
Blair	every 6 months for 2 years, then yearly	CT, MRCP, EUS
Antoñanzas	6–12 months	EUS/MRI/CT
Date	6 months	CT/MRI alternating
Marsoner	6 months	
Ridtitid	3–12 months	CT, MRI, and/or EUS
Hirono	6 months	CT/MRI, tumor markers
Miyasaka	3–6 months	CT, MRI/MRCP, tumor markers
Yamaguchi	3 months (for 2 years, 6 months thereafter)	Not specified
Kwon	At 1, 3, and 6 months, then yearly	CT
He	every 6 months for 2 years, then yearly	CT/MRCP/EUS
Ohtsuka	6 months	CT/MRI alternating
Fujii	6 months	CT or EUS
Niedergethmann	1 year	CT/MRI
Rodriguez	1 year	US/CT/MRI

**Table 3:** Recurrence data following resection for IPMN among included studies.

Author	Median Follow-Up Time (months)	Overall Recurrence Rate (%)	Median (or Mean **) Time to Recurrence (months)
Marchegiani	58	9	17
Al Efishat	42	22	28
Marchegiani	56	18.5	12
Pflüger	114	15	54
Amini	48.9	27.6	84 **
Li	38.5	9.6	8
Dhar	36	10.3	22
Jang	37.8	3.2	46.5
Yogi	46.4	17	20.4
Xourafas		16	59.4
Kang	44.4	5.4	47.4
Winner	32	9.7	21.9
Passot	33.3	20.2	56.5
Yokoyama	60	5	41.6
Chari	36	8	40
Hirono	54.2	5.8	24
D’Angelica	32	9.375	20
Fujii	47	4.9	77.7
White	40	7.7	22
Blair	68		34
Antoñanzas	92.4	5.5	46
Pea		19	27
Ridtitid	53.9	6.8	21.5 **
Hirono	53.5	5.81	12.2
Yamaguchi	27.6	6.7	13.2
Kwon	25.3	4.55	17
Sahora	63		13
Sahora K	60	8.5	34
He	38	17	46
Ohtsuka	64	15.4	23
Park	38.4	1.5	8
Lubezky	50	8	24
Rodriguez	46	7	34.7 **

## Data Availability

Available at request.
